# Negative Effects of Butachlor on the Growth and Physiology of Four Aquatic Plants

**DOI:** 10.3390/plants13020304

**Published:** 2024-01-19

**Authors:** Yixuan Huang, Suting Zhao, Ling Xian, Wei Li, Cunyu Zhou, Junyao Sun

**Affiliations:** 1 School of Horticulture and Gardening, Yangtze University, Jingzhou 434025, China; 2 Aquatic Plants Research Center, Wuhan Botanical Garden, Chinese Academy of Sciences, Wuhan 430074, China; 3 Hubei Key Laboratory of Big Data in Science and Technology, Wuhan Library, Chinese Academy of Sciences, Wuhan 430071, China; 4 Research Center for Ecology, College of Science, Tibet University, Lhasa 850000, China; 5 Hubei Key Laboratory of Wetland Evolution & Ecological Restoration, Wuhan Botanical Garden, Chinese Academy of Sciences, Wuhan 430074, China

**Keywords:** macrophytes, tolerance, herbicide, photosynthetic characteristics, growth characteristics

## Abstract

The increasing use of herbicides in intelligent agricultural production is driven by the time-consuming nature of manual weeding, as well as its ephemeral effectiveness. However, herbicides like butachlor degrade slowly and can be washed away by rainwater, ultimately flowing into the farm ponds and posing risks to aquatic plants. To identify and recommend superior restoration strategies that effectively address the challenges posed by butachlor, we investigated the impacts of butachlor on the growth and physiology of four common aquatic plants (i.e., *Hydrilla verticillata*, *Ceratophyllum demersum*, *Potamogeton maackianus*, and *Myriophyllum aquaticum*) and their potential role in mitigating environmental damage by reducing residual herbicide levels. Our findings indicated that *M. aquaticum* was tolerant to butachlor, exhibiting higher growth rates than other species when exposed to various butachlor concentrations. However, the concentration of butachlor negatively impacted the growth of *H. verticillata*, *C. demersum*, and *P. maackianus*, with higher concentrations leading to more significant inhibitory effects. After a 15-day experimental period, aquatic plants reduced the butachlor residuals in culture mediums across concentrations of 0.5 mg/L, 1 mg/L, and 2 mg/L compared to non-plant controls. Our findings classified *P. maackianus* as butachlor-sensitive and *M. aquaticum* as butachlor-tolerant species. This investigation represents novel research aimed at elucidating the contrasting effects of different concentrations of butachlor on four common aquatic species in the agricultural multi-pond system.

## 1. Introduction

The widespread adoption of herbicides has revolutionized modern farming practices, offering effective and efficient solutions to manage weed infestations and maximize crop yields [1]. However, the extensive use of herbicides such as glyphosate, atrazine, 2,4-D, bentazone, and butachlor raises concerns about their potential environmental impacts and associated risks to ecosystems and human health [2]. Widely used herbicides have been reported to be released into various water bodies [3,4,5]. The transportation of herbicides from agricultural lands to surface and groundwater facilitates their ingress into farm ponds [6,7]. Consequently, herbicides pose a significant risk of contaminating non-target organisms (e.g., aquatic biota) within the aquatic ecosystems owing to their persistence [8]. This persistence prolongs their presence in the environment, increasing the potential for unintended exposure and adverse effects on aquatic biota [9].

Butachlor (N-(butoxymethyl)-2-chloro-2′,6′-diethylacetanilide) has gained widespread recognition as an efficacious and selective acetamide herbicide, resulting in its extensive use in rice cultivation [10]. The effectiveness of butachlor in targeting weeds while minimizing harm to the rice crop has made it a preferred choice among farmers seeking efficient weed management strategies [11]. Moreover, its favorable safety profile and economical value further contribute to its extensive utilization in rice production systems [10]. The study conducted by Liu [12] provides valuable insights into the optimal application concentration for butachlor in rice paddy water, which has been determined to be 4.80 mg/L. This recommended concentration has been carefully determined, considering the dual objectives of achieving effective weed control while minimizing potential environmental impacts. However, the prevalent usage of butachlor nonetheless raises concerns regarding its potential environmental impacts, including its persistence in soils, potential leaching into groundwater, and the risk of non-target organism exposure within the affected ecosystem [13]. The effects on weeds caused by butachlor varies in terms of plant species, plant organs, phenological period, exposure concentration, and duration [14,15]. The use of butachlor poses challenges to the health and diversity of plant communities. Butachlor not only targets weeds but can also damage crops and native plants, with a significant risk of phytotoxicity that may cause stunting, wilting, or death in non-target species exposed to the herbicide [15,16,17].

Different plant species display a range of sensitivities to butachlor, with some species being more susceptible, suffering growth inhibition or other detrimental impacts even at reduced concentrations. Kumar and Jagannath [18] found in a controlled experiment that within the spectrum of modest butachlor concentrations (ranging from 0.15 to 1.00 mg/L), an incremental augmentation in butachlor dosage is concomitantly linked to the inhibition of wheat somatic cell mitosis to diverse extents. One study revealed that butachlor exposure at 5.00 mg/L significantly reduces the fresh weight of Italian ryegrass, showing a clear dose–response relationship with increased concentrations [15]. Butachlor’s absorption and translocation can vary among different plant parts, resulting in a range of effects. Numerous studies have clearly outlined the toxic effects of butachlor on various terrestrial plants [19,20,21]. Ateeq found a potent inhibitory impact of butachlor upon the root tip spindle formation in Allium, resulting in pronounced alterations to root tip morphology, particularly manifesting at a concentration of 5.13 mg/L with an EC50 value [22]. Furthermore, the application of butachlor within anaerobic conditions has manifested a notable 27% reduction in the stem length of rice seedlings, accompanied by corresponding reductions in root and shoot length [23]. Butachlor is absorbed by plant roots, exerting herbicidal effects belowground and causing indirect toxicity to aboveground tissues via root damage [15].

The extensive utilization of butachlor has led to its pervasive presence not only in terrestrial environments but also in aquatic ecosystems [24] Butachlor can be transported to nearby surface water or infiltrate into groundwater through processes such as runoff and leaching [25], which can result in the presence of butachlor residues in aquatic ecosystems [26]. Butachlor have toxic effects on different aquatic organisms, including fish [27], invertebrates [28], algae [29], and aquatic plants [14]. Previous study has predominantly focused on amphibians, fish, algae, and soil microorganisms, leaving aquatic plants with limited and incomplete investigations [27,29]. Aquatic plants are often primary drivers of productivity within aquatic ecosystems, and in many instances can play a role in remediating the chemical contamination in polluted water bodies [30]. Aquatic plants may assimilate butachlor, thus potentially serving as valuable biological indicators for monitoring herbicide pollution and in turn allowing comprehensive ecological risk assessments in aquatic ecosystems to be conducted [9]. Several previous studies examined how herbicides affect different aquatic plants. One study discovered that higher densities of floating plants could capture more herbicides, reducing the amount entering the aquatic environment [31]. Zhou et al. investigated the toxicity of flurochloridone on *Ceratophyllum demersum* and *Lemna minor*, revealing that the herbicide hindered growth and physiological functions, especially at higher concentrations [32]. Some researchers found the combined pollutants of microplastics and glyphosate together had a more detrimental impact on *Salvinia cucullata* than either stressor alone, reducing growth rate, altering morphology, impairing photosynthesis, and increasing oxidative stress [33]. However, few studies have focused on the effect of specific herbicide butachlor on the aquatic plants. Our study specifically assessed four aquatic plant species, including *Hydrilla verticillata*, *Ceratophyllum demersum*, *Potamogeton maackianus*, and *Myriophyllum aquaticum*, regarding growth and physiological changes under butachlor stress. Our research questions were as follows: (1) How do different aquatic plants respond to butachlor in terms of growth and physiology? (2) How sensitive are aquatic plants to butachlor?

By evaluating the tolerance levels of aquatic plant species to butachlor, we can identify suitable candidates capable of thriving in herbicide-contaminated environments. This selection process provides options for cultivating aquatic plants in areas affected by herbicide pollution.

## 2. Results

### 2.1. Effects of Different Butachlor Loadings on the Growth Characteristics of Aquatic Plants

**Figure 1:**
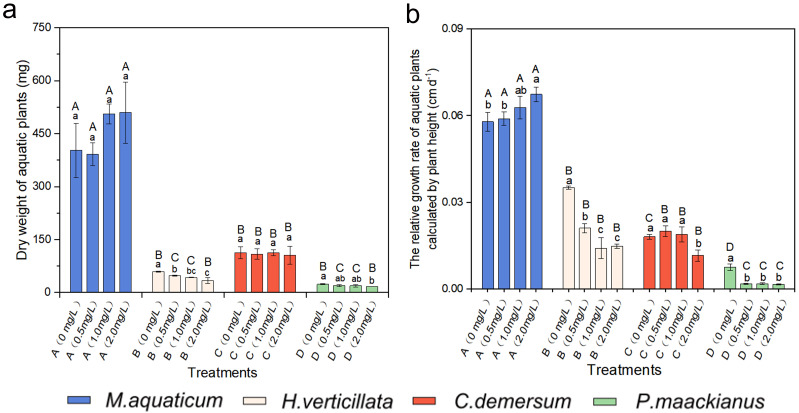
(**a**) Dry weight of aquatic plants; (**b**) The variation in the 15-day relative growth rate calculated by plant height of four aquatic plant species subjected to different concentrations of butachlor stress. The species are abbreviated as follows: A—*M. aquaticum*, B—*H. verticillata*, C—*C. demersum*, and D—*P. maackianus*. The use of lowercase letters within the column indicates statistically significant differences in the growth characteristics of the same species under various butachlor concentrations (*p* < 0.05). The use of uppercase letters indicates the statistically significant differences among plants at the same concentration.

No significant difference was observed in the dry weight of *M. aquaticum* and *C. demersum* under different concentrations of butachlor treatment, as demonstrated in Figure 1a. Conversely, the dry weight of *P. maackianus* showed inhibition at butachlor concentrations exceeding 2.00 mg/L. Likewise, a marked suppression of the dry weight was noted in *H. verticillata* when the concentration of butachlor surpassed 0.50 mg/L.

The impact of various concentrations of butachlor treatment on the relative growth rate based on plant height of different plant species is evident. The species of *M. aquaticum* exhibited a significantly higher relative growth rate in the experimental group compared to the control group (*p* < 0.05, Figure 1b). Moreover, an upward trend in the relative growth rate was observed with increasing concentrations of butachlor. Conversely, the experimental groups of *H. verticillata* and *P. maackianus* demonstrated lower relative growth rates than their respective control groups after treatment with different concentrations of butachlor. Notably, the relative growth rates of these species decreased as the concentration of the butachlor solution increased (*p* < 0.05, Figure 1b). No change was observed in the relative growth rate at concentrations of 0.50 mg/L and 1.00 mg/L of *C. demersum*, but a significant decrease was observed at 2.00 mg/L following treatment with different concentrations of butachlor.

The two-way ANOVA analyses (see Table A1 in Appendix A) demonstrated that species exert a statistically significant influence on plant growth characteristics (*p* < 0.001). Furthermore, a statistically significant interaction was observed between the effects of butachlor loadings and species (*p* < 0.01). However, it was noted that butachlor loadings significantly affected plant relative growth rate, whereas their impact on dry weight was not statistically significant (*p* > 0.1).

### 2.2. Effects of Different Butachlor Loadings on the Photosynthetic Characteristics of Aquatic Plants

**Figure 2:**
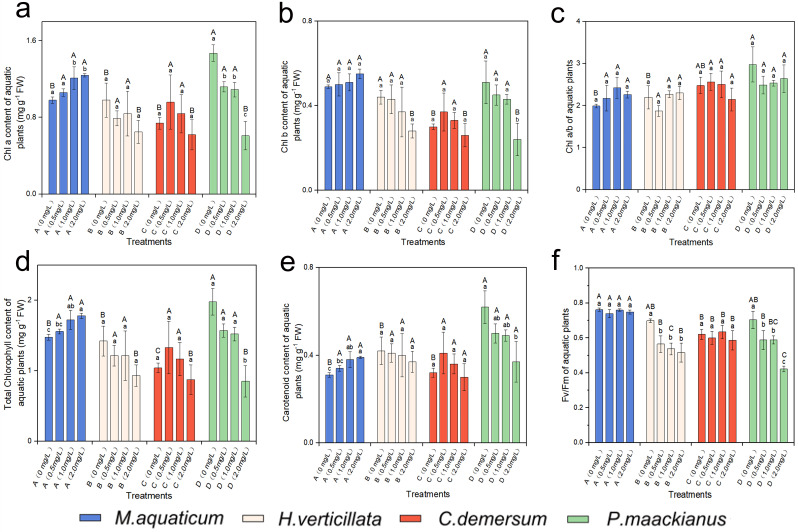
The variations in the following parameters of four aquatic plant species under different concentrations of butachlor stress over a 15-day period: (**a**) Chla, (**b**) Chlb, (**c**) Chl a/b ratio, (**d**) total chlorophyll content, (**e**) carotenoid content, and (**f**) Fv/Fm value. The species are abbreviated as follows: A—*M. aquaticum*, B—*H. verticillata*, C—*C. demersum*, and D—*P. maackianus*. The use of lowercase letters within the column indicates statistically significant differences in the photosynthetic characteristics of the same species under various butachlor concentrations (*p* < 0.05). The use of uppercase letters indicates the statistically significant differences among plants at the same concentration.

The two-way ANOVA analysis revealed a statistically significant effect of butachlor loadings on most plant photosynthetic attributes, with the chl a/b ratio and carotenoid content being the exceptions (Table A2 in Appendix A, Figure 2c,e). It can be observed that *M. aquaticum* exhibited a significant increase in chla content when the butachlor concentration exceeded 1.00 mg/L (Figure 2a). Conversely, *P. maackianus* showed a significant decrease in chla content when the butachlor concentration surpassed 0.50 mg/L, and this inhibition enhanced with increasing butachlor concentration. The content of chlb significantly decreased in *P. maackianus* when the butachlor concentration exceeded 2.00 mg/L, yet the other three species did not show any significant changes at the same butachlor concentrations (Figure 2b).

The results indicated the variations in total chlorophyll among different aquatic plant species under varying butachlor loadings (Figure 2d). The experimental groups of *M. aquaticum* demonstrated significantly higher total chlorophyll compared to the control group across different butachlor concentrations. In contrast, the chlorophyll content of *P. maackianus* exhibited a significant decrease with increasing butachlor concentration (*p* < 0.05, Figure 2d) when subjected to different butachlor loadings.

Figure 2f illustrates the Fv/Fm values obtained from experiments conducted on the species of *H. verticillata* and *P. maackianus* following treatment with different concentrations of butachlor. In comparison to the control group, the experimental group exhibited lower Fv/Fm values for both species (*p* < 0.05, Figure 2f). Additionally, the Fv/Fm values showed a declining trend with increasing butachlor concentration, indicating a dose-dependent decrease in photosynthetic efficiency. Notably, *P. maackianus* was more severely inhibited than *H. verticillata* under the effect of butachlor. Conversely, no significant difference was observed in the Fv/Fm values between the experimental and control groups of *M. aquaticum* suggesting a relatively higher tolerance to butachlor-induced stress.

A statistically significant interaction was observed between the impacts of butachlor loadings and species on photosynthetic characteristics (*p* < 0.001), with the notable exception of carotenoid content (Table A2 in Appendix A).

### 2.3. Decrease of Butachlor at Different Concentrations with Aquatic Plants

**Figure 3:**
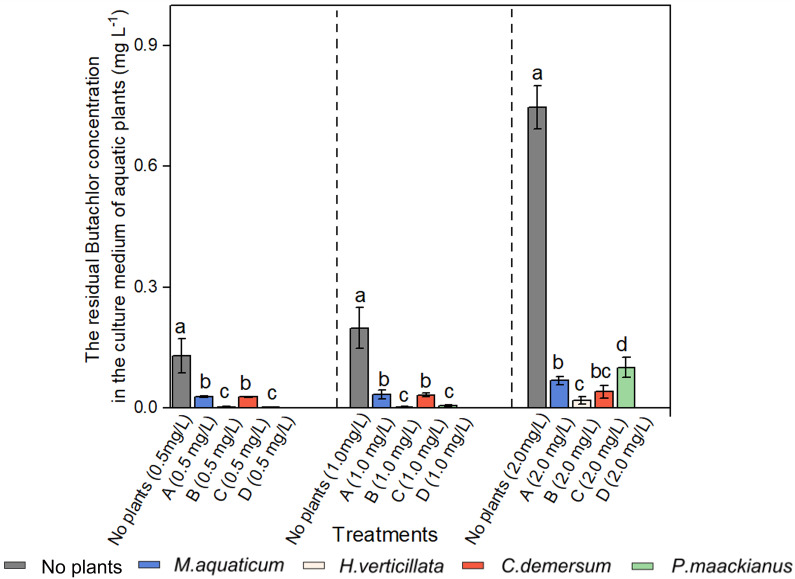
Changes of residual butachlor concentration in the culture medium of blank control group and four aquatic plant species subjected to different concentrations of butachlor stress. The label abbreviations are explained as follows: No plants—the blank control without the addition of plants, A—*M. aquaticum*, B—*H. verticillata*, C—*C. demersum*, and D—*P. maackianus*. The use of lowercase letters within the column indicates statistically significant differences in the residual butachlor concentration of the same species under various butachlor concentrations (*p* < 0.05).

Based on the findings depicted in Figure 3, it is evident that following a 15-day experimental period, in varying concentrations of butachlor (0.5 mg/L, 1 mg/L, and 2 mg/L), the residual butachlor concentration in the culture medium of all aquatic plant experimental treatments was lower than that in treatments with no plants. Comparative analysis among different aquatic plants revealed that when treated with 0.50 mg/L and 1.00 mg/L butachlor, the residual butachlor concentrations in the solution of *H. verticillata* and *P. maackianus* were considerably lower compared to *M. aquaticum* and *C. demersum* (*p* < 0.05, Figure 3). This outcome suggests that *H. verticillata* and *P. maackianus* exhibited the most pronounced efficacy in diminishing the butachlor concentration in the water sample. Furthermore, under the 2.00 mg/L butachlor treatment, the residual butachlor concentration in the solution of *H. verticillata* was significantly lower than in other aquatic plant solutions. Notably, the solution of *P. maackianus* displayed the highest residual butachlor concentration (*p* < 0.05, Figure 3).

**Figure A1:**
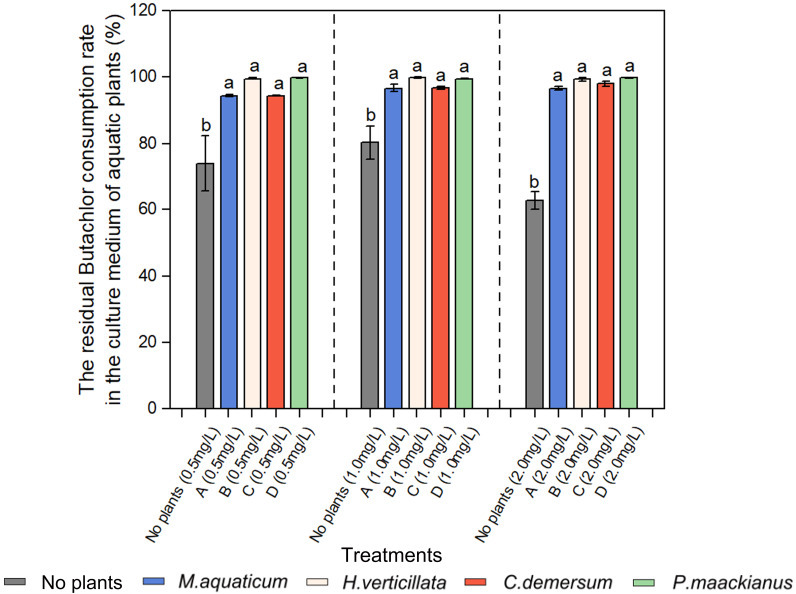
Change of butachlor decay rate in the culture medium of the blank control group and four aquatic plant species subjected to different concentrations of butachlor stress. The label abbreviations are explained as follows: No plants—the blank control without the addition of plants, A—*M. aquaticum*, B—*H. verticillata*, C—*C. demersum*, and D—*P. maackianus*. The use of lowercase letters within the column indicates statistically significant differences in the residual butachlor consumption rate of the same species under various butachlor concentrations (*p* < 0.05).

Concurrently, we calculated the rate of butachlor attenuation using the initial butachlor concentration at the outset and the final concentration at the end of the experiment with. Our observations revealed that the butachlor attenuation rate in the group lacking plant presence was limited to a range of 60–80%. In contrast, the experimental group containing plants exhibited a butachlor attenuation rate exceeding 90% (for more information, see Figure A1 in Appendix A).

## 3. Discussion

### 3.1. The Growth and Physiology of Four Aquatic Plants in Response to Different Butachlor Concentrations

While the broad usage of herbicides across farmland is a well-established occurrence, and there are studies suggesting that the entry of butachlor into water bodies could negatively impact macrophyte communities [15], limited attention has been given to differentiating between butachlor-tolerant and butachlor-sensitive aquatic plants. In this current study, we used Fv/Fm and the total chlorophyll content as key indicators in ascertaining the butachlor tolerance level of aquatic macrophytes. As the concentration of butachlor increased, we observed a reduction in the chlorophyll fluorescence and the relative growth rate of *H. verticillata*, *C. demersum*, and *P. maackianus*. Butachlor would hinder the growth of most aquatic plants, with higher concentrations leading to more pronounced inhibitory effects.

The chlorophyll fluorescence parameter Fv/Fm can be applied to examine biotic and abiotic stress within aquatic plants [34]. Measuring Fv/Fm is critical in identifying potential stressors that could affect the functioning of photosystem II in plants [35]. Stress affects ATP and NADPH production in plants, reducing available reaction centers and causing changes in the Fv/Fm ratio [36]. Fv/Fm is usually steady (0.6–0.8) among species under normal conditions but drops sharply when exposed to stress [36,37]. The photosynthetic pigments of high plants are the material basis for photosynthesis, and chlorophyll content can reflect the growth status and photosynthetic capacity of plants [38]. Our study found that at 0.50 mg/L butachlor, the photosynthetic efficiency (Fv/Fm) of *H. verticillata* and *P. maackianus* was below the stress threshold (Fv/Fm < 0.6). This was accompanied by symptoms like leaf chlorosis and yellowing, reduced chlorophyll content, and lower relative growth rate, signaling that the plant was undergoing stress. Increased butachlor concentrations further affected *P. maackianus*, with higher chlorophyll and carotenoid levels and more significant growth rate reductions than in *H. verticillata*. This greater impact may be due to the characteristics of the species [39]. This result is corroborated by prior research, which indicates that when *Vallisneria natans* is exposed to glyphosate-induced stress, the initial observed response includes a decline in the Fv/Fm value. This is followed by leaf yellowing, a subsequent reduction in chlorophyll content, and slowed plant growth, ultimately leading to the potential death of the plant [40].

Relative growth rate was generally effective in elucidating the effect of the stress on the species assessed [41], with *C. demersum* appearing less sensitive to butachlor than *H. verticillata* and *P. maackianus*. However, the relative growth rate of *C. demersum* was inhibited at 2.00 mg/L of butachlor. Our study supports the existing evidence of herbicides’ negative effects on aquatic plants, for example, the study by Pan [14], which found negative impacts of butachlor on the relative growth rate of submerged plants *C. demersum*, *V. natans*, and *Elodea nuttallii*. Similarly, Chattopadhyay [42] observed butachlor’s inhibitory effects on the relative growth rate of various aquatic plants, with the strongest impact being on *Ottelia alismoides*. Coyner [43] also reported significant reductions in *Potamogeton pectinatus* growth when exposed to chlorsulfuron. They observed a decrease in height (76%), leaf number (50%), and stem number (50%) in plants exposed to 0.25 μg/L chlorsulfuron. A study conducted in Brazil on the chemical management of native and invasive aquatic plants with herbicides glyphosate and saflufenacil revealed high susceptibility of *Eichhornia crassipes* to these treatments; moreover, the combination of both herbicides replicated the effects of glyphosate alone on *E. crassipes* and *Pistia stratiotes* [44].

It is noteworthy that *M. aquaticum* exhibits significantly higher dry weight, and relatively higher growth rates than other species when exposed to various butachlor concentrations, suggesting that *M. aquaticum* is more capable of withstanding butachlor’s presence. This outcome aligns with past research, which has demonstrated that invasive species tend to be more vulnerable to the herbicide flurpyrauxifen-benzyl compared to their native counterparts [45,46]. Under natural conditions, the Fv/Fm ratios of the four studied species were between 0.6 to 0.8. However, upon exposure to high butachlor levels, all except for *M. aquaticum*, saw a sharp drop in Fv/Fm, particularly *P. maackianus*. While there were species-dependent differences in the total chlorophyll content and relative growth rate under natural conditions, only *C. demersum* and *M. aquaticum* did not show significant changes after butachlor treatments, while the other species exhibited a significant decrease in these parameters. Consequently, our findings classify *P. maackianus* as butachlor-sensitive and *M. aquaticum* as butachlor-tolerant, aligning with previous studies [47].

### 3.2. The Tolerance of Aquatic Plants to Different Butachlor Concentrations

The residual concentrations of butachlor in all plant experiment groups’ culture medium were noticeably lower than those in plant-free treatments across various butachlor concentrations. This reduction may be attributed to the metabolic activities in aquatic plants, which potentially accelerate the breakdown and volatilization of herbicides [48,49,50]. Further research is necessary to determine whether the plants absorb butachlor and to elucidate the underlying mechanisms of its uptake. Indeed, we observed that butachlor decomposes faster in the presence of *M. aquatium* [51]. Although plants are damaged after being treated with butachlor, they retain limited biological activity. Previous studies have demonstrated that antioxidant enzymes of the plants decrease oxidative harm induced by non-biological stress, though increasing and prolonging butachlor’s concentration or treatment duration could compromise the antioxidant system [52,53]. This conclusion of the terrestrial plants supports the results of *P. maackianus*, in which the photosynthesis of the plant weakens and growth is inhibited as the concentration of butachlor increases. Upon reaching a butachlor concentration of 2.00 mg/L, the plant’s oxidative system experienced a collapse, rendering the plant unable to withstand such levels. Consequently, this treatment resulted in the highest residual butachlor content among all groups in the experiment (*p* < 0.05, Figure 3).

The solutions containing *H. verticillata* and *P. maackianus* had lower residual butachlor concentrations compared to *M. aquaticum* and *C. demersum* (*p* < 0.05, Figure 3). This suggests that *M. aquatium* and *C. demersum* exhibit higher tolerance to butachlor. The plausible mechanisms for this tolerance among aquatic plants to butachlor may include, firstly, *M. aquatium* and *C. demersum* potentially metabolizing butachlor into less toxic or non-toxic forms through the production of enzymes that can degrade the herbicide. These enzymes could participate in processes such as hydrolysis, oxidation, or conjugation with other molecules, ultimately rendering butachlor less harmful to the plant [54]. Secondly, butachlor typically disrupts specific target sites or enzymes involved in plant growth and development. *M. aquatium* and *C. demersum* may harbor genetic mutations or variations in these target sites, reducing the herbicide’s efficacy in inhibiting the plant’s normal physiological processes [55,56]. Lastly, *M. aquatium* and *C. demersum* exposed to herbicides like butachlor might enhance their antioxidant defenses [57], enabling them to mitigate the oxidative stress induced by the herbicide since herbicides often generate reactive oxygen species (ROS) within plant cells [58]. In summary, the tolerance of various plants to different butachlor concentrations may vary and is speculated to be associated with intrinsic mechanisms within the species. Presently, there is limited research on the effects of herbicides on aquatic plants. Drawing from the toxic effects of herbicides on terrestrial plants, it is reasonable to hypothesize that antioxidant systems within aquatic plants might also help regulate external stress, granting plants some level of herbicide tolerance. However, this tolerance isn’t constant, and high herbicide concentrations can weaken or eliminate the plant’s protective antioxidant system. Our ongoing study targets the tolerance mechanisms, specifically the role of the antioxidant system, in aquatic plants exposed to herbicides like butachlor. Importantly, these mechanisms can vary across different species, with some using multiple strategies at once.

## 4. Material and Methods

### 4.1. Plant Material and Experimental Setups

This study aimed to investigate the impact of butachlor on the growth and physiological characteristics of four aquatic plant species: *H. verticillata*, *C*. *demersum*, *P*. *maackianus*, and *M. aquaticum*. The selection of these specific plant species as indicator species was based on their wide distribution in freshwater ecosystems. The indoor experiment was carried out at the laboratory in Wuhan Botanical Garden, Chinese Academy of Sciences, located at a latitude of 30°55′ N and a longitude of 114°43′ E, over a duration of almost one month from July to August 2022. Throughout the experiment period, the indoor environmental conditions were maintained at an average temperature of 30 ± 4 °C. The light intensity, crucial for photosynthetic processes, was measured using a TriOS RAMSES light quantum meter from Rastede, Germany, yielding an average value of 146 ± 8 μmol m^2^/s.

Approximately 100 mature individuals for each study aquatic species were collected within the Wuhan Botanical Garden. Upon transferring the plant specimens to the laboratory, any adhering algae and soil particles were removed. The four aquatic plant species were individually introduced into separate plastic tanks (top diameter × bottom diameter × height, 61 cm × 45 cm × 65 cm, capacity: 420 L). After one week of acclimation, 24 healthy plants of each species exhibiting consistent biomass were carefully selected and transplanted into a separate polyethylene vessel (top diameter × bottom diameter × height, 17.30 cm × 12.30 cm × 13.40 cm, capacity: 2 L) for the following experiment.

### 4.2. Preparation of Butachlor Solution and Butachlor Treatments

**Figure 4:**
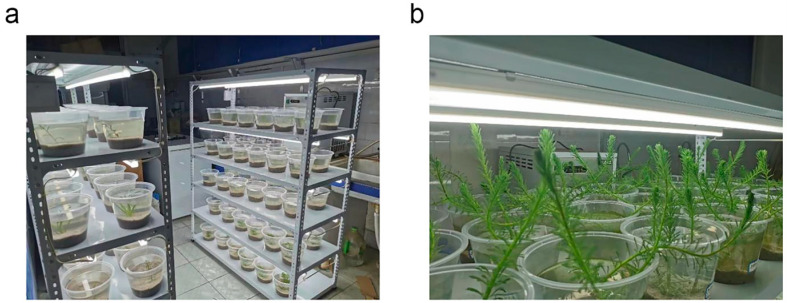
The study aquatic plants and experimental setup. (**a**) Schematic representation of experimental setup, (**b**) Treatment of *M. aquaticum* (photo captured on day five of experiment initiation).

Each of the four plant species was subjected to four treatment (i.e., 0 mg/L (control), 0.50 mg/L, 1.00 mg/L, and 2.00 mg/L) concentrations of butachlor. To ensure robust statistical analysis, each treatment was replicated six times, employing six plastic vessels as parallel treatments. The plants were cultivated in individual vessels containing varying concentrations of butachlor mixed with 10% Hoagland solution. A layer of 3 cm high quartz sand was added to each bowl as a substrate. Stem segments measuring approximately 10~15 cm in height were used (with fresh weights of 170 mg for *P. maackianus*, 450 mg for *H. verticillata*, 1700 mg for *C. demersum*, and 1800 mg for *M. aquaticum*) to ensure consistency in the experiments. The allocation of the 96 bowls within the experimental setup followed a random arrangement, as depicted in Figure 4. The entire experiment lasted 15 days. Chlorophyll fluorescence measurements were conducted on the 1st and 15th days after treatment initiation to assess the photosynthetic performance of plants. Additionally, on the 15th day, chlorophyll content, and the residual butachlor concentration in the culture medium were simultaneously determined. Simultaneously, the second control group devoid of plants was established, with butachlor concentrations of 0.50 mg/L, 1.00 mg/L, and 2.00 mg/L, to investigate the potential impact of plant presence on the decrease of butachlor.

### 4.3. Determination of Growth Characteristics

To examine the impacts of butachlor loadings on the development of aquatic plants, initial growth attributes including leaf height and dry weight were assessed at the end of the experiment. We carefully removed the plants from their culture containers. After rinsing the individual plant, we placed the plants on absorbent paper and covered them with additional absorbent paper to remove any excess surface moisture. The plant height was calculated by a ruler. Then, we dried the plants samples in an oven at 80 °C for 48 h [59] and measured the dry weight for each individual plant by the micro-analytical balance. The relative growth rate (RGR) quantifies the increase of a quantity per unit of time relative to its original value, offering valuable insights into growth dynamics over a specific period under butachlor stress [60]. It is calculated using the following formula:

RGR = (ln(**Q**2) − ln(**Q**1))/(**t**2 − **t**1) (1)

In the Formula (1), **Q** represents the quantity being measured, which was expressed in terms of plant height in this study. The time interval between the two measurements is denoted as **t**, and it can be expressed by days (d) in this study.

### 4.4. Determination of Photosynthetic Characteristics

The maximum quantum yield of chlorophyll fluorescence was assessed (Fv/Fm) using a chlorophyll fluorescence meter (PAM-2100, Walz, Effeltrich, Germany) according to the method used by Apudo et al. [61] We ensured representative sampling by selecting three mature leaves from the top portion of the plant. To prepare the leaves for measurement, a controlled dark adaptation period of 15 min was implemented to stabilize the photosynthetic systems and minimize the effects of previous light exposure.

The total chlorophyll and carotenoid content of the plant samples were determined by spectrophotometry following standardized protocol [62]. Briefly, after extracting from approximately 0.02 g fresh leaves with 5 mL 95% ethanol under dark for 24 h, the content of the photosynthetic pigments was obtained from the absorbance at wavelengths of 665 nm, 649 nm, and 470 nm using a spectrophotometer (TU1810PC, Beijing Purkinje General Instrument Co., Ltd., Beijing, China).

### 4.5. Determination of Butachlor Concentration in the Remaining Solution

The concentration of butachlor in the remaining solution was ascertained by employing a gas chromatography method [63]. We took a 5 mL sample and transferred it into a centrifuge tube. To extract the butachlor, we added 5 mL of n-hexane to the sample and vigorously shook it for 5 min. The resulting mixture was then centrifuged, and the supernatant was carefully transferred to another centrifuge tube. To remove the solvent and concentrate the analyte, we employed a solvent evaporation workstation, allowing the extracted solution to evaporate to dryness. Subsequently, we reconstituted the dried residue with 1 mL of n-hexane. The reconstituted sample was thoroughly shaken for 1 min to ensure proper mixing. To remove any particulate matter or impurities, the reconstituted sample was filtered, and 2 mL of the filtered solution was injected into a sample bottle. The sample bottle was then placed into a gas chromatograph (Agilent 7890B, Santa Clara, CA, USA) for measurement.

### 4.6. Statistical Analysis

To investigate the impacts of various butachlor loading treatments applied during the phase spanning from 1st to 15th day, we employed the one-way analysis of variance (ANOVA) method to examine the differences in plant growth and physiological characteristics. Specifically, we focused on parameters such as the relative growth rate, Fv/Fm, total chlorophyll, and carotenoid content, considering the different levels of butachlor loadings. To determine the statistical significance of the observed differences between the treatments, we employed Tukey’s significance test. To present the obtained results, we utilized the mean ± standard error (mean ± SE) format. A two-way ANOVA was conducted to investigate the influence of butachlor loadings and species on plant growth and physiological parameters. The analysis incorporated two categorical variables: varying butachlor loadings and distinct species classifications. This approach aimed to determine the presence of any statistically significant interactions between the impact of butachlor loadings and species variation on the observed plant characteristics. All statistical analyses were conducted using the Origin 2019 (Northampton, MA, USA) and SPSS 20.0 (SPSS Inc., Chicago, IL, USA) software.

## 5. Conclusions

This study evaluates the varying effects of butachlor concentrations on aquatic species to identify effective restoration methods. We observed that butachlor negatively impacts photosynthesis, growth, and chlorophyll fluorescence of aquatic plants, with harmful effects increasing with concentration. While *M. aquatium* showed resilience, *C. demersum*, *H. verticillata*, and *P. maackianus* were vulnerable to butachlor. Our findings also suggest that aquatic plants can potentially help degrade butachlor. This knowledge aids understanding of the impacts of herbicides and can guide the development of sustainable practices dedicated to preserving pond ecosystems and maintaining water quality. This study not only sheds light on the complex interactions between herbicides and aquatic life but also calls attention to the broad ecological consequences of chemical usage in agriculture. These insights stress the importance of adopting more holistic environmental management practices that encompass the protection of vulnerable species, the exploitation of natural bioremediation processes, and the overall maintenance of ecological balance. Such strategic measures are instrumental in ensuring the sustainability and health of aquatic ecosystems, which are essential for biodiversity and provide numerous ecological services.

## Data Availability

The data that support the findings of this study are available from the corresponding author upon reasonable request. The data are not publicly available due to [privacy or ethical restrictions].

## Appendix A

**Table A1:** Two-way ANOVA for the different growth characteristics for the butachlor loadings and species (* *p* < 0.05, *** *p* < 0.001).

Source of Variation	Dry Weight			Relative Growth Rate		
	*df*	*SS*	*F*	*df*	*SS*	*F*
Butachlor loadings	3	6461	1.38	3	0.0002	12.2 ***
Species	3	1,449,467	310.8 ***	3	0.022	1130 ***
Butachlor × Species	9	31,534	2.25 *	9	0.0009	16.0 ***

**Table A2:** Two-way ANOVA for the different photosynthetic characteristics for the butachlor loadings and species (* *p* < 0.05, ** *p* < 0.01, *** *p* < 0.001).

Source of Variation	Chl a			Chl b			Chl a/b		
	*df*	*SS*	*F*	*df*	*SS*	*F*	*df*	*SS*	*F*
Butachlor loadings	3	0.49	5.7 **	3	0.09	5.5 **	3	0.18	0.7
Species	3	1.07	12.3 ***	3	0.24	14.9 ***	3	2.00	8.2 ***
Butachlor × Species	9	1.11	4.3 **	9	0.11	2.2 *	9	0.92	1.3
**Source of Variation**	**Total Chlorophyll**			**Carotenoid Content**			**Fv/Fm**		
	***df***	***SS***	***F***	***df***	***SS***	***F***	***df***	***SS***	***F***
Butachlor loadings	3	0.98	6.1 **	3	0.03	1.92	3	0.09	17.4 ***
Species	3	2.19	13.4 ***	3	0.16	11.5 ***	3	0.25	44.3 ***
Butachlor × Species	9	1.85	0.002 **	9	0.09	2.26 *	9	0.08	5.22 ***
